# The Antimicrobial Peptide Octopromycin Suppresses Biofilm Formation and Quorum Sensing in *Acinetobacter baumannii*

**DOI:** 10.3390/antibiotics12030623

**Published:** 2023-03-21

**Authors:** Dinusha Chathurangi Rajapaksha, Shan Lakmal Edirisinghe, Chamilani Nikapitiya, Ilson Whang, Mahanama De Zoysa

**Affiliations:** 1 College of Veterinary Medicine and Research Institute of Veterinary Medicine, Chungnam National University, Daejeon 34134, Republic of Korea; 2 National Marine Biodiversity Institute of Korea (MABIK), 75, Jangsan-ro 101 beon-gil, Janghang-eup, Seochun-gun 33662, Chungchungnam-do, Republic of Korea

**Keywords:** *Acinetobacter baumannii*, biofilm, multidrug resistant, octopromycin, quorum-sensing

## Abstract

*Acinetobacter baumannii* is an opportunistic bacterial pathogen that causes severe infections in immunocompromised individuals. *A. baumannii* forms biofilm and produces extracellular matrix, which supports bacteria to survive under harsh conditions and be resistant to antibacterial treatments. In the present study, we investigated the biofilm and quorum-sensing inhibitory effects of antimicrobial peptide, octopromycin in *A. baumannii*. Field emission-scanning electron microscopy results clearly showed significantly reduced biofilm mass and caused a collapse in biofilm architecture at the minimum inhibitory concentration (50 µg/mL) and minimum bactericidal concentration (200 µg/mL) of octopromycin. Antibiotic-resistant persister cells of *A. baumannii* were successfully killed by octopromycin treatment, and it inhibited violacein production in *Chromobacterium violaceum* in a concentration-dependent manner. Octopromycin also inhibited alginate production, surface movements (swarming and swimming), and twitching motility of *A. baumannnii*, confirming its anti-quorum-sensing activity. Multiple metabolic pathways, two-component regulation systems, quorum-sensing, and antibiotic synthesis-related pathways in *A. baumannii* biofilms were strongly affected by octopromycin treatment. The collective findings indicate that the antibacterial peptide octopromycin may control *A. baumannii* biofilms through multi-target interactions. Octopromycin could be a desirable therapeutic option for the prevention and control of *A. baumannii* infections.

## 1. Introduction

*Acinetobacter baumannii* is a short, round, rod-shaped, Gram-negative, opportunistic pathogen [1]. *A. baumannii* has also been included in the six highly virulent and antibiotic-resistant bacteria groups referred to as “ESKAPE” (*Enterococcus faecium*, *Staphylococcus aureus*, *Klebsiella pneumoniae*, *A. baumannii*, *Pseudomonas aeruginosa*, and *Enterobacter* species) pathogens, which are major causes of nosocomial infections [2]. The World Health Organization has designated *A. baumannii* as a critical priority pathogen that poses a great threat to human health [2]. The pathogenesis of *A. baumannii* infections involves multiple virulence factors, including porins, capsules, cell wall lipopolysaccharide, enzymes, biofilm production, motility, and iron-acquisition systems. *A. baumannii* is highly contagious, causes pneumonia, septicemia, meningitis, infections of the urinary tract, wound infections, and it is associated with high mortality [3].

Biofilms are complex communities of microbes attached to a surface or that may form aggregates without adhering to a surface. Biofilms formed by pathogenic bacteria can allow the persistence of bacterial infections. Bacterial biofilm formation involves the surface adhesion of floating (planktonic) bacteria, followed by the formation of a community that is covered with a matrix of exopolysaccharide (also termed extracellular polymeric substances, EPS) [4]. Approximately 65% of all bacterial infections are associated with bacterial biofilms [5]. Biofilms have been linked to various human diseases that include cystic fibrosis [6], dental plaque [7], wound and urinary infections [8], prosthetic joint infections [9], and cardiac valve infections [10]. One major problem posed by biofilms is the increased tolerance to antimicrobial agents, which impairs the treatment of biofilm-related infections [11,12]. The causes of increased antibiotic resistance in bacterial biofilms are not fully understood. Multiple factors implicated in protecting biofilm bacteria from antibiotics include limited antibiotic penetration, horizontal gene transfer, reduced growth rate, persister cells, efflux pumps, and protection by the EPS [13].

Bacterial responses within biofilms are regulated by cellular signals known as quorum-sensing (QS). It is a type of cell-to-cell communication of bacteria that regulates their gene expression in response to cell population density via small signaling molecules. Bacterial QS is operated via various signals, including N-acyl homoserine lactones (AHLs), oligo peptides, furanosyl borate, and hydroxyl-palmitic acid methylester [14]. QS AHLs and oligopeptides have been identified in Gram-positive [15] and Gram-negative bacteria [16]. Many natural compounds with QS inhibitory activities have been identified, which discourage the QS-dependent infections produced by pathogenic bacteria [17]. Several reports described the inhibition of QS in *A. baumannii* by the antibacterial peptide Cec4 [12] and the chemical compound virstatin [18]. However, currently available antibiofilm drugs are not completely effective against *A. baumannii* biofilms.

Antimicrobial peptides (AMPs) are components of the innate immune system across all kingdoms that are less likely to trigger drug resistance [19]. AMPs have characteristic properties that include a positive (cationic) charge, amphiphilic (hydrophilic and hydrophobic) nature, and low molecular weight (<10 kDa). These properties facilitate rapid permeabilization of AMPs through microbial membranes [20]. AMPs have been discovered in almost all organisms and display marked functional and structural diversities. In addition to direct antimicrobial activity, AMPs may have immunomodulatory and wound-healing functions, which can be exploited in their use as novel therapeutics [21]. Furthermore, AMPs may inhibit biofilms by disrupting the membrane potential or altering the membrane permeability of the biofilm bacteria, interrupting cell signaling (QS or motility), degrading EPS, inhibiting the stringent response inhibition, and down-regulating target genes [22]. Therefore, AMPs are considered as novel drugs for the control of bacterial biofilms and associated infections [23].

In our previous study, we identified a novel amino acid sequence with AMP characteristics in the proline-rich protein 5 gene of *Octopus minor*. The selected amino acid sequence with features of AMPs was termed “Octopromycin”. It has a positive charge (+5), a high hydrophobic residue ratio (36%), and a predicted alpha-helix secondary structure. Octopromycin has shown minimum inhibitory concentration (MIC) and minimum bactericidal concentration (MBC) of 50 and 200 µg/mL, respectively, for a lab strain of *A. baumannii* [24]. These levels of octopromycin inhibited and eradicated *A. baumannii* biofilms.

The mechanism of action of octopromycin in biofilm inhibition is unclear. In the present study, the inhibitory effects of octopromycin on biofilm and the QS properties of *A. baumannii* were evaluated. The biofilm inhibitory activity of octopromycin was determined using the minimum biofilm inhibitory concentration (MBIC) and the minimum biofilm eradication concentration (MBEC). Morphological alterations and persister cell density were examined to determine the mode of action of octopromycin against *A. baumannii* biofilms. The QS inhibition of octopromycin was confirmed using a bioreporter strain, *Chromobacterium violacium*. Subsequently, the production of the alginate component of EPS production and inhibition of surface and twitching motility were determined in *A. baumannii*. The findings implicate octopromycin as a prospective drug for controlling multidrug resistant (MDR) *A. baumannii* biofilms.

## 2. Results

### 2.1. Octopromycin Inhibits and Eradicates A. baumannii Biofilms

We reported the preliminary data related to the antibiofilm activities of octopromycin against *A. baumannii* in our previous study [24]. There was no complete inhibition or eradication of *A. baumannii* biofilm at MIC (50 µg/mL) and MBC (200 µg/mL) levels [24]. To determine the MBIC and MBEC, a higher concentration range (0–800 μg/mL) of octopromycin was treated with *A. baumannii*. Octopromycin displayed inhibitory and eradication effects on *A. baumannii* biofilm, with an MBIC ranging from 350–500 μg/mL and an MBEC ranging from 600–700 μg/mL (data not shown). At the MBC of 200 μg/mL, the eradication level of mature biofilms exceeded 60%, which was consistent with our previous results [24].

### 2.2. Octopromycin Destroys A. baumannii Biofilm Structure

**Figure 1:**
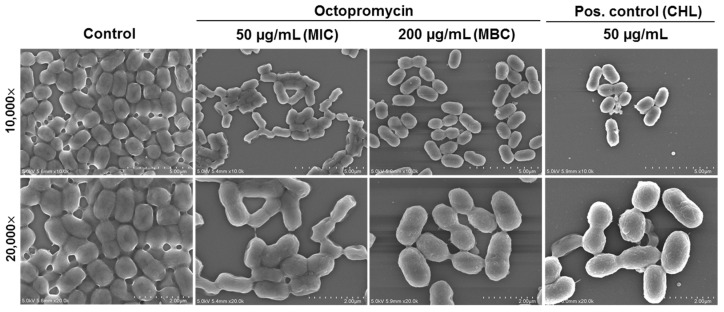
Field emission scanning electron microscopy (FE-SEM) visualization for inhibitory activity of octopromycin against *A. baumannii* biofilms. From left to right, the representative lower magnification (10,000×) and higher magnification (20,000×) images show control (phosphate buffer saline (PBS)-treated), octopromycin at MIC (50 μg/mL), and MBC (200 μg/mL), and chloramphenicol (CHL) at 50 μg/mL as positive (Pos.) control. *A. baumannii* were incubated on glass slides for 24 h, followed by fixation with 2.5% glutaraldehyde and dehydration in a series of alcohol solutions prior to examination of microstructure.

*A. baumannii* colonizes the surfaces of medical equipment and indwelling medical devices, including urinary catheters. The resulting biofilms are the basis of long-term and recurrent infections in patients [25]. In the present study, coverslips were used as in vitro surfaces for biofilm formation. The effects of octopromycin on biofilm structure and removal were determined by field emission scanning electron microscopy (FE-SEM) imaging (Figure 1). After 24 h of culturing, *A. baumannii* in the control (untreated) group adhered mostly to the coverslip and the cell membranes were intact, forming a typical “mushroom cloud” three-dimensional structure. After treatment with octopromycin at MIC (50 µg/mL), MBC (200 µg/mL), and chloramphenicol (CHL) as a positive control, biofilm structure and bacterial density were considerably inhibited. Moreover, some cell surfaces appeared to be deformed, and morphometric alterations were observed upon octopromycin treatment. Interestingly, in the octopromycin (200 µg/mL) and positive control (CHL 50 µg/mL) groups, a similar disappearance of extracellular matrix was evident, with the presence of individual bacteria.

### 2.3. Inhibitory Effect of Octopromycin on Persister A. baumannii and Alginate Production

**Figure 2:**
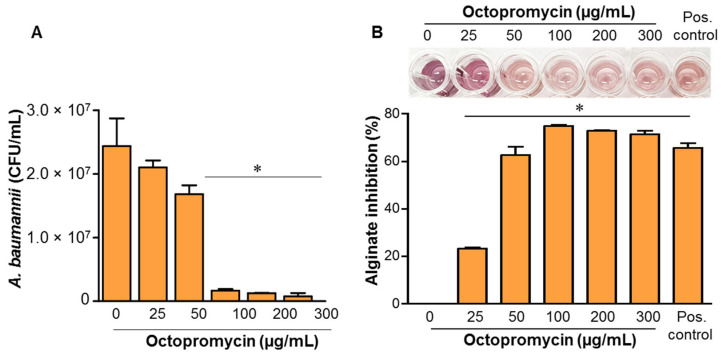
Inhibitory effect of octopromycin on persister cells and alginate production in *A. baumannii*. (**A**) Inhibition of persister cells was determined using serial dilutions of octopromycin (0−300 µg/mL). Each concentration was established in persister cell suspensions that were incubated at 25 °C for 12 h. The number of viable bacteria was determined microbiologically. (**B**) Effect of octopromycin on alginate production was determined using the same series of octopromycin dilutions. *A. baumannii* treated with 100 μg/mL of CHL was used as a positive (Pos.) control. * *p* ≤ 0.05 compared to the control (untreated) group. The error bars indicate mean ± standard deviation (*n* = 3).

The effect of octopromycin on *A. baumannii* persisters was confirmed using agar plating. *A. baumannii* persisters were killed in a dose-dependent manner by octopromycin; more than half of the persisters were significantly (*p* ≤ 0.05) eliminated by concentrations of octopromycin ≥2 × MIC (100 µg/mL). The results implicated the peptide as a potent candidate for killing persister cells (Figure 2A). Alginate is an EPS component that is important in biofilm structure. It acts as an intercellular material that is required for the formation of thicker three-dimensional biofilms. Alginate production is controlled by QS. In the present study, alginate was extracted from octopromycin-treated and untreated cultures of *A. baumannii*. Quantitative analysis revealed that octopromycin decreased *A. baumannii* alginate production in a concentration-dependent manner (Figure 2B). Octopromycin resulted in a significant (*p* ≤ 0.05) reduction (23–74%) in alginate production at concentrations ranging from 25–300 μg/mL. These results confirm that octopromycin inhibits biofilm formation by killing persister cells and reducing alginate production, which are essential for the protection of the bacteria in adverse surroundings.

### 2.4. Octopromycin Inhibits QS in C. violaceum

**Figure 3:**
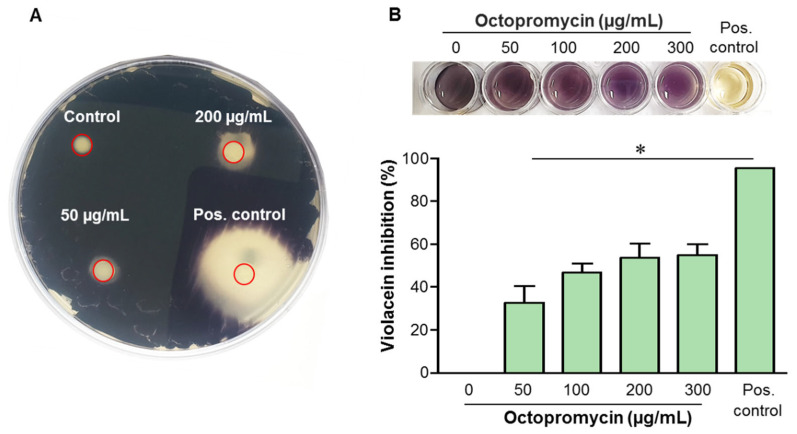
Inhibition of violacein production by octopromycin (**A**) Quorum-sensing bioassay results. The plate shows representative results of inhibition of violacein production by octopromycin at MIC (50 µg/mL), MBC (200 µg/mL), and positive (Pos.) control (CHL 100 μg/mL). The absence of purple pigment formation was considered to indicate a potential QS inhibitor. (**B**) Representative series of images of violacein production by *C. vialaceum* treated with 0–300 µg/mL of octopromycin by the microtiter plate method. The bar graph presents the quantitative results of the inhibition of N-[3-oxohexanoyl]-l-homoserine lactone (OHL)-mediated violacein production. * *p* ≤ 0.05 compared to the control group. The error bars indicate mean ± standard deviation (*n* = 3).

The QS inhibitory effect of octopromycin was assessed by the loss of the purple pigment produced by violacein in *C. violaceum*. Octopromycin inhibited violacein production in a concentration-dependent manner at all tested concentrations. A clear inner zone indicated the inhibition of bacterial growth by octopromycin (Figure 3A). Non-transparent halo zones around the discs indicated the inhibition of violacein production, which may be due to interference with the induced production of AHLs. As the octopromycin concentration increased, the size of the halo zones around the discs increased. Quantitative determination of violacein inhibition was performed using the microtiter plate (MTP) assay. Octopromycin at all tested concentrations (50–300 µg/mL) significantly (*p* ≤ 0.05) decreased the violacein production (Figure 3B). At the lowest concentration, the violacein inhibition level was 32.54% compared to the negative control. The inhibitory activity was gradually enhanced with octopromycin treatment, reaching a maximum of 54.75% at a concentration of 300 μg/mL.

### 2.5. Octopromycin Decreases Surface and Twitching Motility of A. baumannii

**Figure 4:**
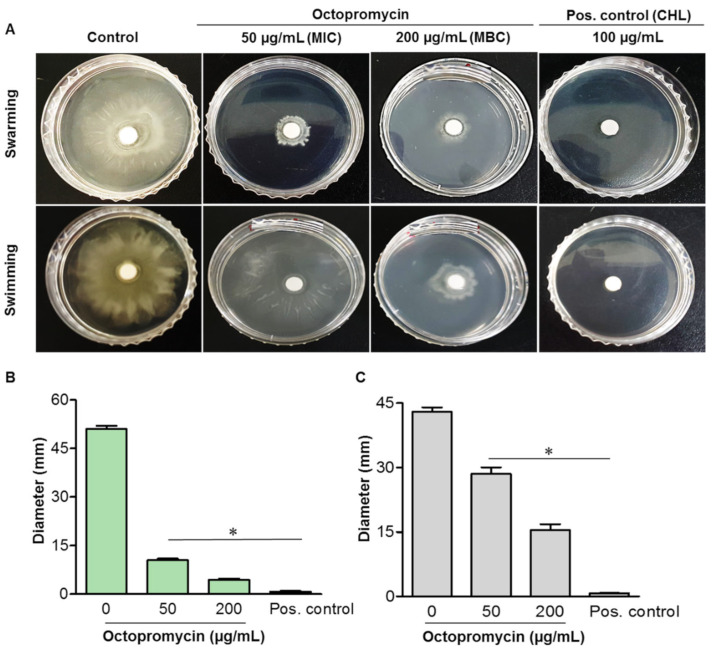
Effect of octopromycin on swarming and swimming motility of *A. baumannii*. Plates were point-inoculated from overnight cultures. Images were taken after 20 h of incubation at 25 °C. (**A**) Swarming and swimming bacteria circles in the presence of octopromycin MIC (50 μg/mL) and MBC (200 μg/mL). Diameter of the (**B**) swarming and (**C**) swimming bacteria circles. Positive (Pos.) control; CHL (100 μg/mL). * *p* ≤ 0.05 compared to the control (untreated) group. The error bars indicate means ± standard deviation (*n* = 3).

Swarming and swimming motility play crucial roles in QS-dependent biofilm formation [26]. Octopromycin-mediated inhibition of *A. baumannii* motility was examined. Octopromycin inhibited both the swarming and swimming behaviors of *A. baumannii*. Higher levels of swarming and swimming inhibition were observed at both tested concentrations (50 and 200 μg/mL). Bacterial motility was suppressed in a dose-dependent manner (Figure 4A). Octopromycin significantly (*p* ≤ 0.05) reduced the swarming and swimming diameters of *A. baumannii* relative to that of the untreated control. At 50 μg/mL, octopromycin decreased the swarming and swimming diameters up to 10.5 and 28.5 mm, respectively, compared to the control (51 and 43 mm, respectively). At 200 μg/mL of octopromycin, swarming and swimming diameters decreased to 4.4 and 15.4 mm, respectively (Figure 4B,C).

**Figure 5:**
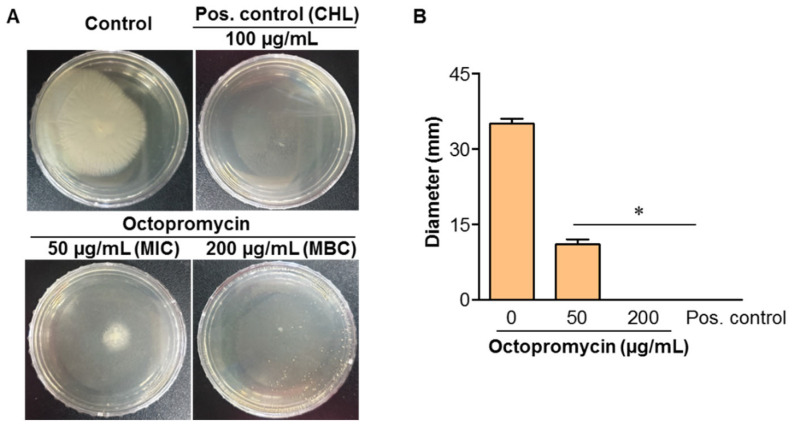
Effect of octopromycin on twitching motility of *A. baumannii*. Single colony of *A. baumannii* was picked from a TSA culture plate, inoculated vertically to the bottom of the plate, and images were taken after 20 h of incubation at 25 °C. (**A**) Twitching motility of *A. baumannii* in the presence of octopromycin MIC (50 µg/mL) and MBC (200 µg/mL). (**B**) Diameter of the twitch motility of *A. baumannii*. Positive (Pos.) control; CHL (100 μg/mL) used as a * *p* ≤ 0.05 compared to the untreated control. The error bars indicate mean ± standard deviation (*n* = 3).

The twitching motility of *A. baumannii* is mediated by the type IV pili. The retraction and extension of pili control biofilm formation at the initial stage [27]. After incubation at 25 °C for 20 h, two different types of colonies were evident. One was on the surface of the medium, and the other was between the plate and the bottom of the TSA plate (data not shown). Octopromycin inhibited twitching motility in a concentration-dependent manner at both tested concentrations (50 and 200 μg/mL). Octopromycin treatment significantly (*p* ≤ 0.05) reduced the twitching motility diameter of *A. baumannii* relative to that of the untreated control (Figure 5A). At 50 μg/mL, octopromycin decreased this diameter compared to the control (11.0 vs. 35.0 mm). At 200 μg/mL, the twitching diameter was 0 mm (colony was absent) (Figure 5B).

## 3. Discussion

The MIC level for antibiotics can be increased by 1000 times or more after bacteria form a biofilm, as described in Macia et al. [28]. Most of the currently available antibiotics are ineffective for treating biofilm-related infections owing to their higher MBIC and MBEC values, which may cause lethal toxicity effects [29]. Thus, it is extremely important to design and screen potential drugs that can effectively minimize and eradicate biofilm-related infections. AMPs can suppress QS and control the initial adhesion of bacteria to the surface, thus preventing biofilm formation [30]. Moreover, they kill bacteria embedded in biofilms or disturb the maturation of biofilms by inhibiting or eradicating them.

Our previous study showed that octopromycin has an antibacterial effect on *A. baumannii* with multiple modes of action associated with ultrastructural and permeability changes and oxidative stress by reactive oxygen species (ROS) generation [24]. Furthermore, octopromycin did not show considerable cytotoxic effects on human (HEK293T) or mouse (Raw264.7) cells at concentrations up to 400 μg/mL and also did not cause hemolysis in mouse red blood cells at high concentrations (<300 µg/mL) [24]. In this study, we first performed MBIC and MBEC assays to determine the effective dose of octopromycin for biofilm-forming *A. baumannii*. Results revealed that the MBIC (350–500 μg/mL) and MBEC (600–700 μg/mL) of octopromycin were comparatively high compared to their respective MICs and MBCs. However, octopromycin at 200 μg/mL eradicated more than 60% of mature biofilms. Weiwei *et al*. [12] reported that AMP Cec4 eradicated the clinical carbapenem-resistant *A. baumannii* biofilm, showing MBIC of 64–128 μg/mL and MBEC of 256–512 μg/mL [12]. However, different modes of action of octopromycin were further explained by interference with the inhibition of QS, cell-to-cell adhesion mechanisms, and disruption of extracellular DNA, proteins, and EPS.

FE-SEM results indicated that untreated *A. baumannii* biofilms could form thick EPS on the glass slide. In the octopromycin-treated groups (50 and 200 μg/mL), biofilms easily detached and the bacterial surfaces were shrunken and damaged. Similar to the present results, an EDTA-containing nanoemulsion extensively destroyed the surfaces of *A. baumannii* within biofilms [31]. Moreover, AMP-Cec4-treated coverslips and urinary catheters were used as carriers to evaluate the ability of removing *A. baumannii* biofilms. At MIC (4 µg/mL), the biofilm structure was destroyed, and small vesicles appeared on the surface of the membrane, leading to cell disintegration [12]. In the present study, the effect of octopromycin on *A. baumannii* biofilm morphological disturbance was confirmed, with an activity similar to that of previously tested antibiofilm agents.

Although some antibiotics penetrate the biofilm matrix, they are not active against dormant subpopulations of persisters [32]. Persisters are bacterial cells that survive in very high antibiotic concentrations regardless of their antibiotic susceptibility. Persisters have been discovered in many bacterial pathogens, including *A. baumannii* [33] and *P. aerogenosa* [34]. Persisters have a permeability barrier that prevents the penetration of chemical compounds, and the bacteria remain in a dormant state [35]. Persister cell development has also been examined in relation to aminoglycosides, cephalosporins, and fluoroquinolones. Polymyxins (polymyxin B and colistin) are last-resort antibiotics used to treat persister-producing MDR pathogens. Interestingly, we observed that octopromycin effectively controlled *A. baumannii* persistence in a concentration-dependent manner. Several tryptophan/arginine-containing AMPs kill persister cells [36]. Moreover, AMP ZY4 combats *P. aeruginosa* and *A. baumannii* persisters [32]. The inhibitory activities of octopromycin on biofilms and persisters confirmed its potential multi-target function.

One of the major factors promoting the success of MDR pathogenic bacteria is the production of a biofilm-enveloping matrix of biomolecules termed EPS, which is primarily composed of polysaccharides, proteins, and deoxyribonucleic acid (DNA). EPS accumulates through active cellular secretion, retention of cell lysis products, and adsorption of external molecules [37]. The viscous exopolysaccharide alginate tends to protect bacteria in adverse environments against the action of human leukocytes and enhances adhesion to surfaces. Davenport et al. [37] reported that *A. baumannii* EPS acts as a “universal protector” by inhibiting tobramycin activity against bacterial cells by limiting antibiotic diffusion to the bottom of the biofilm. In our study, octopromycin inhibited the alginate production by *A. baumannii* in a concentration-dependent manner, confirming its strong activity. Similar to our results, previous studies have reported that alginate production in *P. aeruginosa* was inhibited by *Matricaria chamonilla* and quercetin [26]. Further studies are required to investigate the inhibitory effects of other components of EPS.

Many opportunistic pathogens, such as *A. baumannii* [38], *Serratia marcescens*, and *P. aeruginosa* [39], control the production of their virulence factors using a QS system. Other than biofilm development, QS signaling is also required to modulate the biofilm phenotype while involving surface mortality and the production of EPS components to develop strong antibiotic resistance [40]. Therefore, inhibition of QS signaling is one of the different strategies used to control biofilm-forming, drug-resistant microbes [41]. Thus, the search for “quorum quenchers”, which refers to the mechanism by which bacterial communication can be disrupted, has recently increased. The QS circuit of *A. baumannii* entails two elements: the AbaI inducer and its cognate receptor AbaR, which are homologous to the typical LuxI/LuxR system found in Gram-negative bacteria [38]. In the present study, we first confirmed the QS inhibitory activity of octopromycin in *C. violaceum*. This is a Gram-negative beta-proteobacterium that produces violet colonies in common laboratory media. These violet pigments are derived from the pigment violacein, encoded by the vio operon, whose expression is regulated by QS. This QS trait of *C. violaceum* is an easily observable and quantifiable marker that is widely used as a model organism for the basic screening of quorum quenchers [42]. In the QS inhibition bioassay, octopromycin exhibited a concentration-dependent reduction in violacein production, as indicated by the non-pigmented zones. In the MTP assay with *C. violaceum*, octopromycin depleted violacein production up to 54.75% at 300 μg/mL. Our results are comparable with those of Gopu et al. [26] who reported violacein inhibition of 83.23% by quercetin at a concentration of 80 μg/mL in *C. violaceum* CECT5999. Vasavi et al. [43] reported a >50% reduction in violacein production at 100 μg/mL of *Centella asiatica*. Alginate is an EPS component that protects the bacteria from adversity in their surroundings and also promotes adhesion to solid surfaces [44]. It acts as an intercellular material that is required for the formation of thicker, three-dimensional biofilms. Thus, good alginate producers such as *P. aeruginosa* and *P. fluorescens* showed enhanced detachment of the bacteria by stimulating the negative anchoring properties [45], and tolerability in low-temperature ranges [44]. Our data showed that >50 µg/mL octopromycin-treated *A. baumannii* cultures had strong inhibition of alginate production (>60%). Therefore, we suggest inhibition of aliginate production by octopromycin is strong evidence for its anti-biofilm function.

Swarming and swimming motility control bacterial biofilm formation via surface attachment and complex signal transduction systems. *A. baumannii* lacks the genes required for flagellar biosynthesis, which is necessary for motility. However, genomic analysis of clinical isolates of *A. baumannii* has revealed the presence of genes (*pilA-C*, *pilF*, *pilM-Q*, *pilW*, *pilZ*, *pilR-T*, and *pilA*) required for type IV pili assembly [27]. Therefore, in semi-solid media, *A. baumannii* still exhibits flagellum-independent motility. Our results showed that octopromycin significantly reduced both the swarming and swimming motility of *A. baumannii* compared with the untreated control. These results are comparable to those of Dong et al. [46] reported significant inhibition of *Escherichia coli* swimming motility by HV2 and PG-1 peptides. Chabane *et al*., compared 30 *A. baumannii* strains that were mobile and found that 60% of *A. baumannii* strains underwent motility treatment with virstatin [18]. In another study [47], cinnamaldehyde reduced biofilm formation in *P. aeruginosa* by inhibiting swarming motility. Although QS has been suggested as a novel therapeutic strategy for new drug development [48], further studies are required to explore the QS inhibition mechanism of *A. baumannii* by octopromycin, which targets biomolecules of the QS network.

In summary, we studied the previously characterized AMP octopromycin concerning its effects on *A. baumannii* biofilm formation and QS. Octopromycin suppressed the biofilm development with MBIC and MBEC in the ranges of 350–500 and 600–700 μg/mL, respectively. The antibiofilm activity of octopromycin against *A. baumannii* involved multiple modes of action, such as morphological and ultrastructural damage to the extracellular matrix, alteration of membrane permeability, and inhibition of persister cell growth. QS inhibition suppresses alginate production and inhibits motility (surface and twitching). Our results provide new insights into the treatment of clinical *A. baumannii* biofilm-related infections and the development of AMPs as new antimicrobial drugs. In order to disclose the detailed inhibition of biofilm formation, octopromycin will be tested for interference with signaling pathways involved in the synthesis of EPS components in our future studies. Such identification of the AMP-based antibiofilm strategies would be required to deal with the prevention criteria of bacterial antibiotic resistance.

## 4. Materials and Methods

### 4.1. Bacterial Identification and Culture Conditions

Laboratory strains of *A. baumannii* and *C. violaceum* were confirmed by amplification of the 16S rRNA gene using the universal primers 27F (5′ AGAGTTTGATCMTGGCTCAG-3′) and 1492R (5′-TACGGYTACCTTGTTACGACTT-3′). *A. baumannii* and *C. violaceum* were cultured in trypticase soy broth/agar (TSB/TSA; BD, Reno, NV, USA) and incubated at 25 °C.

### 4.2. Determination of MBIC and MBEC

To determine the biofilm inhibitory effect of octopromycin, a previously described method was used with slight modifications [12]. Briefly, 200 µL of the bacterial culture (1 × 10^6^ CFU/mL) was inoculated into a 96-well plate. All plates were incubated at 25 °C for 24 h. Then, the culture medium was removed, and the wells were washed three times with PBS to remove planktonic bacteria. Next, TSB medium (200 µL) containing different concentrations of octopromycin (0–800 µg/mL) was added to each well. TSB medium without octopromycin was used as a negative control. All plates were incubated at 25 °C for another 24 h. The lowest concentration without bacterial growth was considered the MBIC. The culture plate was washed with PBS, and 200 µL of TSB medium was added to each well. The plate was incubated at 25 °C for another 24 h to allow the surviving biofilm bacteria to regrow. The lowest concentration without bacterial growth was considered the MBEC.

### 4.3. FE-SEM Analysis

To visualize the biofilm inhibitory activity of octopromycin on *A. baumannii*, FE-SEM imaging was performed as previously described, with slight modifications [24]. Briefly, 50 and 200 µg/mL of octopromycin (MIC and MBC, respectively) or CHL (50 µg/mL) were added to the wells of a 6-well microplate containing polylysine-treated sterile cover glass. Two milliliters of the cell suspension at a density of 1 × 10^6^ CFU/mL was added to the wells. After incubation at 25 °C for 24 h, the cover glass was gently removed and fixed in 2.5% glutaraldehyde at 25 °C for 1 h. The prefixed biofilms were washed with PBS and dehydrated using serial concentrations of ethanol (30, 50, 70, 80, 90, and 100%). The fixed cells were dried and coated with platinum using an ion sputter device (E-1030, Hitachi, Tokyo, Japan). *A. baumannii* cells from all groups were observed by FE-SEM (Sirion FEI, Eindhoven, The Netherlands).

### 4.4. Effect of Octopromycin on Inhibition of A. baumannii Persisters

The activity of octopromycin against *A. baumannii* persister cells was determined according to a previously described method [32]. Briefly, *A. baumannii* was cultured to the exponential phase and diluted to 1 × 10^6^ CFU/mL in fresh culture broth (TSB). One hundred microliters of this suspension was transferred to a 96-well plate and incubated at 25 °C for 24 h. Planktonic bacteria were removed by washing three times with sterile PBS. Next, 100 μL of fresh medium containing CHL (MBC 50×) was added to the wells and incubated for another 24 h under the same conditions. After incubation, planktonic bacteria were removed by washing three times with PBS, and adherent persisters were dislodged in 100 μL of fresh PBS. Serial dilutions of octopromycin (0–300 µg/mL) were added to each persister suspension and incubated at 25 °C for 12 h. The number of viable bacteria was determined by the standard agar plating method. PBS was used as negative control, and the experiments were conducted in triplicate.

### 4.5. QS Inhibition Bioassay in C. violaceum

*C. violaceum* is a Gram-negative bacterium that forms the pigment violacein, which is regulated by the vio operon [42]. The QS inhibitory potential of octopromycin was investigated using a laboratory strain of *C. violaceum*, as described by Gopu *et al*., [26] with slight modifications. Overnight grown cultures of *C. violaceum* were swabbed evenly on TSA plates supplemented with a 5 μM QS signal, N-[3-oxohexanoyl]-l-homoserine lactone (OHL; Sigma-Aldrich, St. Louis, MO, USA). Twenty microliters of octopromycin at MIC (50 μg/mL) and MBC (200 μg/mL) levels were loaded on the sterile discs, dried, placed on the agar plates, and incubated at 25 °C. The plates were observed for violacein inhibition, which was evident as obscure, colorless areas around the discs. Sterilized distilled water and CHL (100 µg/mL) were used as the negative and positive controls, respectively.

Violacein inhibition by octopromycin in *C. violaceum* was quantitatively analyzed using the MTP method. Briefly, 1% of the overnight grown culture was inoculated into TSB supplemented with 10 μM OHL and plated in a 96-well microtiter plate. Wells were added with octopromycin at different concentrations (0–300 μg/mL), incubated at 25 °C for 24 h, and extracted for violacein pigment [26]. One milliliter of culture from each well was centrifuged at 1500× *g* for 5 min to precipitate violacein. The obtained pellet was dissolved in dimethyl sulfoxide (1 mL) and vortexed robustly to completely solubilize violacein. The mixture was centrifuged to remove the bacterial cells and quantified at 585 nm using a microplate reader (Bio-Rad, Hercules, CA, USA). The experiment was repeated in triplicate. The percentage of inhibition was calculated using the following formula:

Violacein inhibition (%) = [(Ab control − Ab peptide)/(Ab control)] × 100

where Ab peptide represents the absorbance value of octopromycin treated and Ab control represents the absorbance of the untreated control.

### 4.6. Effect of Octopromycin on Alginate Production in A. baumannii

The effect of octopromycin on the disruption of biofilms due to the inhibited production of low molecular weight alginate oligosaccharide was determined as described by Gholami et al. [49] with some modifications. Briefly, a 1% overnight culture of *A. baumannii* was subcultured in fresh TSB that was not supplemented or supplemented with octopromycin (0–300 μg/mL). The inoculated broths were incubated overnight at 25 °C in a shaker incubator (180 rpm). After incubation, alginate production was estimated. Six hundred microliters of boric acid-sulfuric acid solution in a 4:1 ratio was slowly added to 70 μL of the test sample and placed in an ice bath. The mixture was vortexed for 10 s and placed in an ice bath. To this mixture, 20 μL of 0.2% carbazole (Sigma-Aldrich, St. Louis, MO, USA) dissolved in ethanol was added and vortexed for 10 s. The mixture was incubated at 55 °C for 30 min. Absorbance was measured at 530 nm using a microplate reader (Bio-Rad, Hercules, CA, USA). The percentage of alginate inhibition was calculated using the following formula:

Alginate inhibition (%) = [(Ab control − Ab peptide)/(Ab control)] ×100

where Ab peptide represents the absorbance value of octopromycin treatment and Ab control represents the absorbance of the untreated control.

### 4.7. Effect of Octopromycin on Swimming, Swarming, and Twitching Motility of A. baumannii

The inhibition of *A. bauamnnii* motility was determined by swimming and swarming motility assays, as described by Gholami et al. [49]. For surface motility assays, *A. baumannii* was point inoculated at the center of swimming agar (1 g tryptone, 0.5 g NaCl, and 0.3 g agar in 100 mL) and swarming agar (1 g peptone, 0.5 g NaCl, 0.5 g agar, and 0.5 g filter sterilized D-glucose in 100 mL). The agar also contained MIC (50 μg/mL) and MBC (200 μg/mL) levels of octopromycin. Inoculated plates were incubated at 25 °C for 24 h. Agar media without octopromycin and CHL (100 µg/mL) were the negative and positive controls, respectively. The twitching motility assay was conducted as described by Weiwei et al. [12] with some modifications. Briefly, twitching plates were made with 0.5% TSA alone and TSA containing 50 or 200 μg/mL of octopromycin. A single colony of *A. baumannii* was picked from a normal TSA agar plate and inoculated vertically at the bottom of the plate to facilitate growth at the intersection of the bottom of the agar and the culture plate. The plates were incubated at 25 °C for 20 h. Agar medium without octopromycin and CHL (100 µg/mL) were used as negative and positive controls, respectively.

### 4.8. Statistical Analysis

Data were analyzed using GraphPad Prism software version 5 (GraphPad Software Inc., La Jolla, CA, USA). Data analysis was performed using one-way and/or two-way analysis of variance (ANOVA) to determine the significance between the experimental groups and/or time points. Bonferroni post hoc and/or unpaired two-tailed t-tests were conducted to compare the average means of the controls and treatments. The cutoff for the significance level was set at *p* ≤ 0.05.

## 5. Conclusions

In this study, we systematically investigated the inhibitory effects of biofilm formation and QS using in vitro assays to reveal the antibiofilm potential of octopromycin against MDR *A. baumannii*. FE-SEM analysis results revealed significant inhibition of biofilm mass, bacterial density, and structure at low doses (50 and 200 µg/mL) of octopromycin. The formation of persister cells in biofilms involves adaptation to environmental changes. Regarding the various virulence factors present in Gram-negative bacteria, QS systems protect against antibiotic pressure to form biofilms. The anti-QS activity of octopromycin was confirmed through violacein production in *C. violaceum* as part of the biofilm inhibition by affecting bacterial cross-talk. Moreover, anti-QS activity was further shown to involve inhibition of alginate production, and swimming, swarming, and twitching motility behaviors in *A. baumannii* treated with octopromycin. The collective findings indicate the potential of octopromycin as an AMP in the inhibition of biofilm formation by pathogenic bacteria.

## Data Availability

Not applicable.
